# Fish rubbings, ‘gyotaku’, as a source of historical biodiversity data

**DOI:** 10.3897/zookeys.904.47721

**Published:** 2020-01-16

**Authors:** Yusuke Miyazaki, Atsunobu Murase

**Affiliations:** 1 Department of Child Studies and Welfare, Shiraume Gakuen College, 1-830 Ogawa-chou, Kodaira, Tokyo 187-8570, Japan; 2 Nobeoka Marine Science Station, Field Science Center, University of Miyazaki, 376-6 Akamizu, Nobeoka, Miyazaki 889-0517, Japan; 3 Department of Marine Biology and Environmental Sciences, Faculty of Agriculture, University of Miyazaki, 1-1 Gakuen-Kibanadai-Nishi, Miyazaki 889-2192, Japan

**Keywords:** biogeography, citizen science, data mining, fish rubbing, red list

## Abstract

Methods for obtaining historical biodiversity information are mostly limited to examining museum specimens or surveying past literature. Such materials are sometimes time limited due to degradation, discarding, or other loss. The Japanese cultural art of ‘gyotaku’, which means “fish impression” or “fish rubbing” in English, captures accurate images of fish specimens, and has been used by recreational fishermen and artists since the Edo Period (the oldest known ‘gyotaku’ was made in 1839). ‘Gyotaku’ images often include distributional information, i.e., locality and sampling date. To determine the extent and usefulness of these data, field and questionnaire surveys targeting leisure fishing and boating stores were conducted in the following regions where threatened or extinct fishing targets exist (four regions including the northernmost to the southernmost regions). As a result, 261 ‘gyotaku’ rubbings were digitally copied with their owners’ consents. From these, distributional data were extracted for 218 individuals, which roughly represented regional fish faunas and common fishing targets. The peak number of ‘gyotaku’ stocked at the surveyed shops was made in 2002, while ones made before 1985 were much fewer. The number of ‘gyotaku’ rubbings made in recent years shows a recovery trend after 2011–2012. The present study demonstrates the validity of examining ‘gyotaku’ for historical biodiversity information.

## Introduction

Access to historical biodiversity information is limited, being mainly obtained via museum specimens, past literature, movies, photographs/images and/or other historical materials such as classic monographs (e.g., Hayashi 2014; Rocha et al. 2014; Schilthuizen et al. 2015). These potential data sources, in particular historical materials, are sometimes lost by deliberate or accidental disposal or, for example, by fire or an estate liquidation where materials are scattered far from their origin. There are other similar examples where biodiversity information can be lost over time: seeds in seed banks can die within decades of their collection (e.g., Telewski and Zeevaart 2002; Nishihiro et al. 2016), and environmental DNA cannot be detected several hours or days after sampling (e.g., Thomsen et al. 2012; Bohmann et al. 2014). Thus, it is important for historical biodiversity information to be accessed and recorded as a matter of urgency. Copying or extracting information from privately owned materials is of highest priority.

Biodiversity observations have been made not only by researchers but also by citizens, even prior to the recent rise of citizen science projects (Miller-Rushing et al. 2012; Kobori et al. 2016). Although data mining from citizens’ observation records is a legitimate method of citizen science (Fink and Hochachka 2012), limitations on data availability have not been well documented except for online data (e.g., Miyazaki et al. 2015).

**Figure 1.**
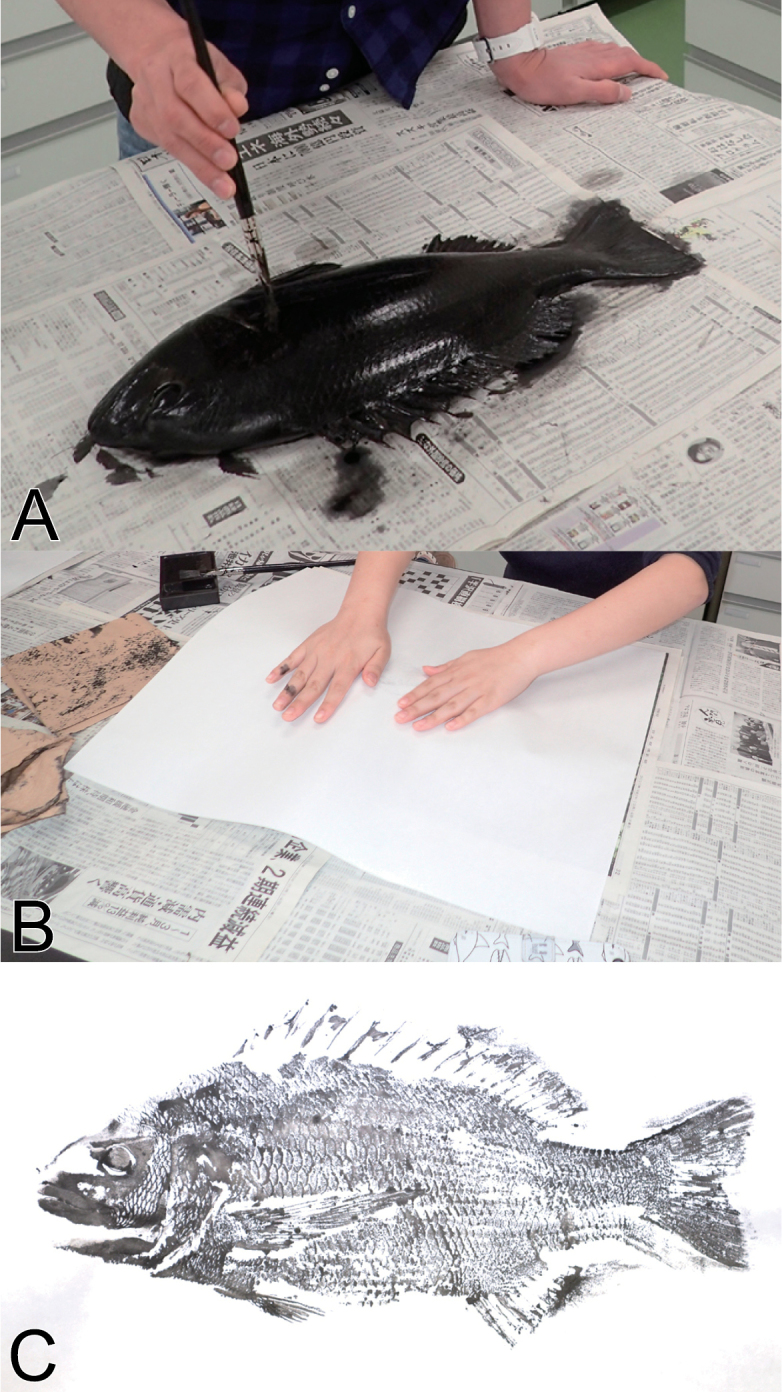
An explanation of fish rubbing (‘gyotaku’, in Japanese). **A** 1^st^ step – the fish specimen is painted using ink **B** 2^nd^ step – the specimen is covered with a sheet of paper **C** the finished image of the fish specimen on the paper. This is known as the direct method of ‘gyotaku’; there is also an indirect method whereby a sheet of paper is placed on the fish specimen, then the sheet is painted by hand using ink. See also Hiyama (1964a, b) and Shimizu (1972, 1975).

In Japan, many recreational fishers have recorded their memorable catches as ‘gyotaku’ (魚拓), which means fish impression or fish rubbing in English (Fig. 1), since the last Edo Period (the current oldest known ‘gyotaku’ dates back to late February 1839) (Hiyama 1964a, b; Shimizu 1975; Nakajima 2005). ‘Gyotaku’ is made directly from fish specimen(s), and usually includes information such as sampling date and locality, the name(s) of the fisher(s), its witness(es), the fish species (frequently its local name), and fishing tackle used. In recent decades, color versions of ‘gyotaku’ have become well developed, and used for art and educational purposes (Hiyama 1964a, b; Shimizu 1972, 1975; Yamamoto 1998; Stokes 2001; Baggett and Shaw 2008). In contrast, the traditional method is printed by using black writing ink. Generally, color prints for art rarely include specimen data including sampling locality and date (e.g., Hiyama 1964a, b; Shimizu 1975; Yamamoto 1998).

We hypothesized that historical biodiversity data attached to ‘gyotaku’ prints are at risk of being lost, and that the number of ‘gyotaku’ prints is generally declining, being replaced with photographs from digital cameras and/or smart phones. The number of fishing-related shops that are personally managed (rather than the large chain stores) and therefore likely to stock original ‘gyotaku’ prints may be decreasing in recent years due to their owners retiring, an increase in chain store numbers, and/or a decrease in recreational fishers (Miyazaki in press).

In the present study, we attempt to validate these hypotheses by collecting data of ‘gyotaku’ from recreational fishing shops where threatened fish species are distributed according to both the national and regional Red Lists. The potential use of ‘gyotaku’ for historical biodiversity information (Miyazaki and Fukui 2018; Miyazaki in press) is discussed.

## Materials and methods

First, preliminary field surveys were conducted at three fishing shops in Miyazaki Prefecture, and one recreational boating shop in Chiba Prefecture where we found ‘gyotaku’ of threatened species were stocked via a reference (Onoue 2004) and by chance during in August 2016 (Miyazaki Pref.), and in March 2017 (Chiba Pref.). Second, we also conducted preliminary surveys of recreational fishing shops at the northernmost and southernmost regions of Japan in order to understand the ‘gyotaku’ information available at the latitudinal limits of Japan (from the subarctic to the tropics). Referencing the *Town Page* (yellow pages by the Nippon Telegraph and Telephone Corporation) of the Souya (northernmost) and the Yaeyama (southernmost) regions, we identified relevant shops (six for Souya in November 2017, and ten for Yaeyama in May 2017). In these surveys, we asked for information on the presence/absence of ‘gyotaku’ and the possibility of photographing them, and other relevant data. Where possible, we photographed ‘gyotaku’ stocks, and sought permission to use the images for research.

Our questionnaires mainly surveyed three regions of Japan where threatened fish species are distributed according to the national Red List (Ministry of the Environment, Japan 2017): the Sakhalin taimen, *Hucho perryi* (Brevoort, 1856) for Hokkaido (234 shops and stores); the small-scale sillago, *Sillago parvisquamis* Gill, 1861 for Tokyo Bay (274 shops and stores); and the Japanese lates, *Lates japonicus* Katayama & Taki, 1984 for Miyazaki Prefecture (80 shops and stores). The Souya region, which had been previously surveyed and no ‘gyotaku’ with distributional data were recorded from there, was excluded from this.

An explanation of the aim of our research and an answer sheet, which covered fifteen items for informed consent based on a research ethics review at the first author’s institution, were attached to the questionnaire. In the surveys conducted during July–September 2018, we asked for information on the presence/absence of ‘gyotaku’, and the possibility of copying relevant data. When possible, we conducted field surveys to photograph ‘gyotaku’ in March 2019.

We pooled the collected ‘gyotaku’ data, and statistically analyzed the dataset by a state space model using R v. 3.6.0 (R Core Team 2019) and the *dlm* package (Petris 2010) that applies a local level model with filtering and smoothing.

## Results and discussion

Of the stores and shops targeted by our second preliminary surveys, none (of six) in the northernmost (Souya) region and three (of ten) in the southernmost (Yaeyama) region stocked ‘gyotaku’ rubbings with distributional information.

Regarding the questionnaires, fourteen surveys were returned unopened due to stores being closed down, and 56 responses were received from others, indicating that the questionnaire response rate was 9.5%. Our field surveys were permitted by nine stores and shops that stocked ‘gyotaku’ rubbings, based on the questionnaire surveys, while 82% of the responses recorded no stock of ‘gyotaku’. This low response rate was possibly caused by us not paying to have the surveys completed and by a high perceived workload to complete the answers.

In total, 261 ‘gyotaku’ rubbings, with 325 printed individual specimens (i.e., a part of ‘gyotaku’ has multiple individuals on a single sheet), were found among the targeted shops (Table 1; Suppl. material 1). All data recorded were integrated to the ‘gyotaku’ database (https://zukan.com/gyotaku/) using the same system as *WEB sakana-zukan* (see Miyazaki et al. 2014). Among the data obtained in the present study, we extracted distributional data for 221 individuals. Distributional data for an additional 14 individuals were obtained through interviewing the holders of the ‘gyotaku’ regarding date and/or locality information, resulting in a total of 235 individuals with distributional data (Table 1). Among the prints, 68 Japanese fish and three cephalopod species were represented, but 65 of these (14.9%) did not include a fish name (Tables 1, 2; Suppl. material 1). In general, a limited number of species are targeted by recreational fishers. Anglers and shop staff accurately identify the main fishing targets, and misidentifications are quickly corrected by other fishers in the local community. A pertinent example is the common octopus, *Octopus vulgaris* Cuvier, 1797, which is identified as a main fishing target by one fishing shop in Yokohama City, Tokyo Bay. ‘Gyotaku’ images of an octopus from this shop is likely to be *O. vulgaris* even if there is no name on the specific ‘gyotaku’. However, this study did not validate such identifications based on external morphology and/or molecular analyses.

**Table 1.** Details of ‘gyotaku’ rubbings surveyed from the shops in the present study.

**Shops surveyed**	**Region**	**Shop style**	**The number of**			
			‘**gyotaku**’	**individuals printed**	**distributional data^1^**	**species (potential)^2^**
A	Hokkaido	Tackles and bait shops	23	23	20	4
B	Hokkaido	Tackles and bait shops	11	11	11	9
C	Hokkaido	Tackles and bait shops	44	53	37 + 8	8
D	Hokkaido	Tackles and bait shops	4	4	3 + 1	4
E	Hokkaido	Tackles and bait shops	1	1	0	1
Sub-total in the Hokkaido area			83	92	71 + 9	18
F	Tokyo Bay	Tackles and bait shops	27	31	31	17
G	Tokyo Bay	Tackles and bait shops	22	23	16 + 3	8
H	Tokyo Bay	Tackles and bait shops	9	9	7 + 1	8
I	Tokyo Bay	Ship shops	39	41	32	7
J	Tokyo Bay	Ship shops	9	15	4	6
Sub-total around the Tokyo Bay area			106	119	90 + 4	32
K	Miyazaki	Tackles and bait shops	12	12	10	4
L	Miyazaki	Tackles and bait shops	8	8	6 + 1	3
M	Miyazaki	Tackles and bait shops	6	6	5	5
Sub-total in the Miyazaki area			26	26	21 + 1	9
N	Yaeyama	Tackles and bait shops	10	11	10	4
O	Yaeyama	Tackles and bait shops	8	8	8	6
P	Yaeyama	Tackles and bait shops	28	69	21	18
Sub-total in the Yaeyama area			46	88	39	21
Total			261	325	221 + 14	68

^1^ Where two numbers are provided (e.g., 37 + 8) the second number refers to data obtained from the owner rather than indicated on the ‘gyotaku’ rubbings. ^2^The number reflects expert opinions provided by the current authors, but more rigorous identifications have not yet been conducted.

**Figure 2.**
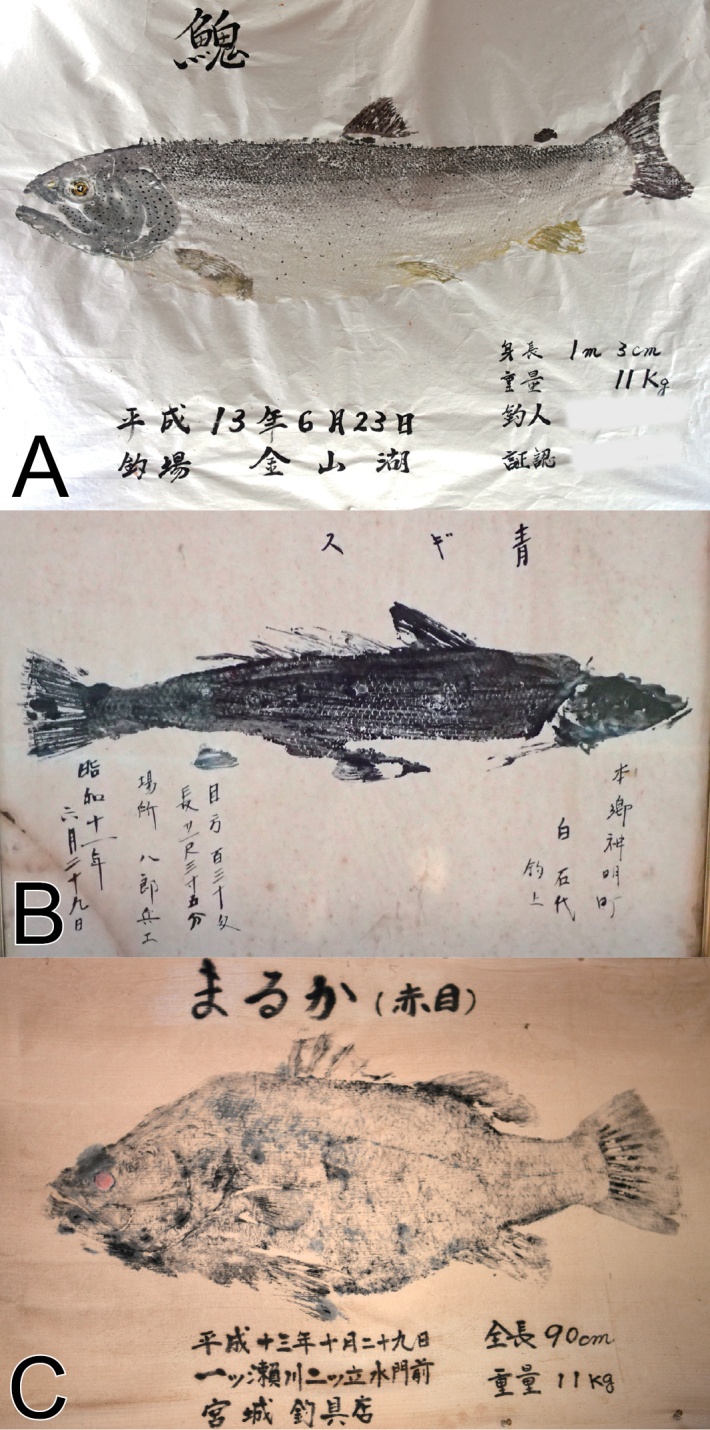
Three species targeted in leisure fishing and listed as threatened species in the Japanese national Red List. **A***Hucho perryi* (Brevoort, 1856) from a shop in Hokkaido **B***Sillago parvisquamis* Gill, 1861 from a shop facing Tokyo Bay **C***Lates japonicus* Katayama & Taki, 1984 from a shop in Miyazaki Prefecture.

The observed species compositions reflected the biogeography of the regions (Table 2; Suppl. material 1). For example, prints of seven individuals of *Hucho perryi* were recorded from only Hokkaido. Similarly, one individual of *Sillago parvisquamis* was recorded from only around Tokyo Bay, while three individuals of *Lates japonicus* were recorded from only Miyazaki Prefecture (Fig. 2). These three species are listed as threatened in national and prefectural Red Lists. In particular, populations of *S. parvisquamis* are probably extinct in Tokyo Bay. The last reliable record from Tokyo Bay is from 1975–1976 (Shigeta and Usuki 2011). Additionally, the populations of *L. japonicus* at Miyazaki Prefecture were listed on the Specified Prefectural Endangered Species of Wild Fauna and Flora on 21 December 2012; this prohibits the capture, holding, receiving, and giving of, and other interactions with, the species without the prefectural governor’s permission (Miyazaki Prefecture 2012; Murase et al. 2019). Given the rarity of these threatened species in some regions, ‘gyotaku’ are probably important vouchers for estimating historical population status, and factors of decline or extinction.

**Table 2.** The composition of the species name given for various individual ‘gyotaku’.

Species	Number of Individual(s)				Remarks
	Hokkaido	Tokyo Bay	Miyazaki	Yaeyama	
Loliginidae					
*Sepia esculenta* Hoyle, 1885	0	0	0	1	
*Sepioteuthis lessoniana* Férussac, 1831	0	1	0	3	
PISCES					
Cyprinidae					
*Carassius cuvieri* Temminck & Schlegel, 1846	0	2	0	0	
*Cyprinus carpio* Linnaeus, 1758	0	2	0	0	
*Tribolodon hakonensis* (Günther, 1880)	1	0	0	0	
Plecoglossidae					
*Plecoglossus altivelis altivelis* (Temminck & Schlegel, 1846)	1	0	0	0	
Salmonidae					
*Hucho perryi* (Brevoort, 1856)	7	0	0	0	
*Oncorhynchus keta* (Walbaum, 1792)	4	0	0	0	
*Oncorhynchus masou masou* (Brevoort, 1856)	18	1	1	0	
*Oncorhynchus mykiss* (Walbaum, 1792)	5	2	0	0	
*Salmo trutta* Linnaeus, 1758	14	0	0	0	
*Salvelinus leucomaenis leucomaenis* (Pallas, 1814)	11	0	0	0	
Sebastidae					
*Sebastes cheni* Barsukov, 1988	0	1	0	0	
*Sebastes schlegelii* Hilgendorf, 1880	1	0	0	0	
*Sebastiscus marmoratus* (Cuvier, 1829)	0	1	0	0	
*Sebastes* sp.	2	0	0	0	‘Soi’ or ‘Mazoi’ in Japanese
Platycephalidae					
*Platycephalus* sp. 2 *sensu* Nakabo (2002)	0	4	0	0	
Serranidae					
*Epinephelus lanceolatus* (Bloch, 1790)	0	0	0	1	
*Niphon spinosus* Cuvier, 1828	0	1	0	0	
*Plectropomus leopardus* (Lacepède, 1802)	0	0	0	2	
Centrarchidae					
*Micropterus salmoides* (Lacepède, 1802)	0	2	0	0	
Lateolabracidae					
*Lateolabrax japonicus* (Cuvier, 1828)	0	7	8	0	
Latidae					
*Lates japonicus* Katayama & Taki, 1984	0	0	3	0	
Carangidae					
*Caranx ignobilis* (Forsskål, 1775)	0	0	0	12	
*Caranx melampygus* Cuvier, 1833	0	0	0	1	
*Caranx* sp.	0	0	0	2	‘Gāra’ in Japanese
*Pseudocaranx dentex* (Bloch & Schneider, 1801)	0	1	0	0	
*Seriola lalandi* Valenciennes, 1833	0	2	0	0	
*Seriola rivoliana* Valenciennes, 1833	0	0	0	1	
Sparidae					
*Acanthopagrus latus* (Houttuyn, 1782)	0	0	2	0	
*Acanthopagrus schlegelii* (Bleeker, 1854)	1	12	6	0	
*Acanthopagrus sivicolus* Akazaki, 1962	0	0	0	2	
*Pagrus major* (Temminck & Schlegel, 1844)	0	3	0	0	
Lethrinidae					
*Lethrinus nebulosus* (Forsskål, 1775)	0	0	0	4	
Branchiostegidae					
*Branchiostegus japonicus* (Houttuyn, 1782)	0	1	0	0	
Oplegnathidae					
*Oplegnathus fasciatus* (Temminck & Schlegel, 1844)	0	2	0	0	
*Oplegnathus punctatus* (Temminck & Schlegel, 1844)	0	2	1	0	
Sciaenidae					
*Argyrosomus japonicus* (Temminck & Schlegel, 1844)	0	1	1	0	
Sillaginidae					
*Sillago japonica* Temminck & Schlegel, 1843	0	1	0	0	
*Sillago parvisquamis* Gill, 1861	0	1	0	0	
Rachycentridae					
*Rachycentron canadum* (Linnaeus, 1766)	0	0	0	1	
Coryphaenidae					
*Coryphaena hippurus* Linnaeus, 1758	0	0	0	3	
Kyphosidae					
*Kyphosus cinerascens* (Forsskål, 1775)	0	0	0	1	
Girellidae					
*Girella leonina* (Richardson, 1846)	0	0	2	0	
*Girella punctata* Gray, 1835	0	8	0	0	
Haemulidae					
*Plectorhinchus cinctus* (Temminck & Schlegel, 1843)	0	0	1	0	
Labridae					
*Cheilinus undulatus* Rüppell, 1835	0	1	0	0	
*Semicossyphus reticulatus* (Valenciennes, 1839)	1	0	0	0	
Scaridae					
*Calotomus japonicus* (Valenciennes, 1840)	0	1	0	0	
Hexagrammidae					
*Hexagrammos otakii* Jordan & Starks, 1895	0	4	0	0	
Siganidae					
*Siganus guttatus* (Bloch, 1787)	0	0	0	2	
Scombridae					
*Katsuwonus pelamis* (Linnaeus, 1758)	0	1	0	0	
*Thunnus albacares* (Bonnaterre, 1788)	0	0	0	1	
Sphyraenidae					
*Sphyraena barracuda* (Edwards, 1771)	0	0	0	2	
Trichiuridae					
*Trichiurus* sp.	0	0	0	1	‘Tachiuo’ in Japanese
Istiophoridae					
*Istiophorus platypterus* (Shaw & Nodder, 1792)	0	0	0	1	
Pleuronectidae					
*Kareius bicoloratus* (Basilewsky, 1855)	1	0	0	0	
*Pleuronectes herzensteini* (Jordan & Snyder, 1901)	1	0	0	0	
*Pleuronectes schrenki* (Schmidt, 1904)	2	0	0	0	
*Verasper moseri* Jordan & Gilbert, 1898	1	0	0	0	
Paralichthyidae					
*Paralichthys olivaceus* (Temminck & Schlegel, 1846)	2	3	0	0	
Monacanthidae					
*Stephanolepis cirrhifer* (Temminck & Schlegel, 1850)	0	4	0	0	
NO NAME GIVEN*	19	55	2	46	

* The individuals have been given no name in the ‘gyotaku’.

Species belonging to other families such as Salmonidae and Pleuronectidae, which originate in cold waters, were mostly recorded from Hokkaido rather than the other surveyed regions (Table 2; Suppl. material 1). On the other hand, several carangid fishes (*Caranx* spp.), the Okinawa seabream (*Acanthopagrus sivicolus* Akazaki, 1962), the spangled emperor [*Lethrinus nebulosus* (Forsskål, 1775)], the orange-spotted spinefoot [*Siganus guttatus* (Bloch, 1787)] and others originating from warm waters were recorded from only the Yaeyama region (Table 2). Another seabream species, *Acanthopagrus latus* (Houttuyn, 1782), which shares a similar distributional pattern with *L. japonicus*, was recorded from only Miyazaki Prefecture. Furthermore, our list (Table 2) also included exotic non-native species, i.e., the rainbow trout, *Oncorhynchus mykiss* (Walbaum, 1792) and the brown trout, *Salmo trutta* Linnaeus, 1758, from the fishing tackle stores at Hokkaido, and *O. mykiss* and the largemouth bass, *Micropterus salmoides* (Lacepède, 1802), from a fishing tackle store in Tokyo Metropolis. Furthermore, some images printed of ‘gyotaku’ were found at stores well outside the pictured species’ known range; generally, these resources had been provided by customers who had traveled to other regions for leisure fishing trips. Overall, the species composition displayed in the ‘gyotaku’ approximately reflected the fish faunas of each biogeographic region.

**Figure 3.**
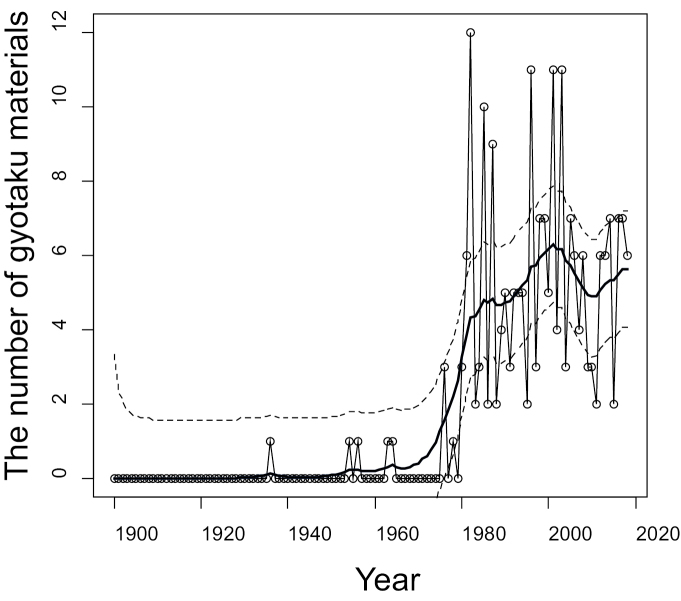
The number of ‘gyotaku’ rubbings stocked at the 16 fishing shops targeted by the present study based on the year of creation. The bold and dotted lines indicate the estimation and the 95% CI (upper and lower limits) based on the statistical analysis using a state space model.

We also obtained a statistically estimated result using a state space model (Fig. 3). This estimation showed very few ‘gyotaku’ available from before 1985, with a peak in 2002. These results suggest that using this technique to gather historical data is valid for perhaps the last 30 years or so and not prior to that. Obtaining useful ‘gyotaku’ more than 30 years old is unlikely. A decline in number was observed during 2011 and 2012, which probably reflects an indirect effect of the catastrophic tsunamis and the nuclear accidents caused by the Great East Japan Earthquake on 11 March 2011 (see also Kataoka 2013). Our data does not support the hypothesis that the use of ‘gyotaku’ will be decreasing over recent years due to the rise of digital photography. We suggest that Japanese recreational fishers may be continuing to use the ‘gyotaku’ method in addition to digital photography to record their memorable catches.

Currently, the oldest ‘gyotaku’ material is a collection of the Tsuruoka City Library made in 1839 (Nakajima 2005). Others from the 19^th^ Century were made in 1850s–1860s and are now collections of the Homma Museum of Art and the Chido Museum (Hiyama 1964a, b; Shimizu 1972, 1975). The oldest material found in the present study was made in 1936, indicating that it is difficult to find very old ‘gyotaku’ rubbings at leisure fishing stores and shops. Storage of ‘gyotaku’ in the public areas of shops and stores is usually less than ideal, with exposure to tobacco smoke, sunlight, and moisture. This is the main reason for deteriorating ‘gyotaku’. In fact, some shop owners reported disposing of older damaged materials. Further field surveys of, for example, museum and private collections are required to discover older ‘gyotaku’ and extract relevant data.

In conclusion, distributional data related to fish diversity records were able to be mined from ‘gyotaku’. However, this method is time limited with respect to data rescue from the general public. The volume of data obtained in this study is too small to analyze statistically from the perspective of ecology, biogeography, or other similar disciplines. Additionally, validation of the identifications sourced from the ‘gyotaku’ is required via taxonomic evaluations. This could be done by examining the external morphology captured in the printed image and possibly by trying to obtain biological material from the print for molecular analysis (i.e., based on the residuum of dried DNA on the sheet). Overall, further research is required into the use of ‘gyotaku’ rubbings for acquiring historical biodiversity data.

## Supplementary materials

Supplementary material 1 The attributional information of the ‘gyotaku’ materials observed by the present study Data type: species data Explanation note: Although the identifications of each individual have not still validated via scientifc evaluations, the scientific names are provisional based on the fish names given by creaters with our suggestions. https://binary.pensoft.net/file/372418This dataset is made available under the Open Database License (http://opendatacommons.org/licenses/odbl/1.0/). The Open Database License (ODbL) is a license agreement intended to allow users to freely share, modify, and use this Dataset while maintaining this same freedom for others, provided that the original source and author(s) are credited.Yusuke Miyazaki, Atsunobu Murase
